# Activated Carbon for CO_2_ Adsorption from Avocado Seeds Activated with NaOH: The Significance of the Production Method

**DOI:** 10.3390/ma17164157

**Published:** 2024-08-22

**Authors:** Joanna Siemak, Grzegorz Mikołajczak, Magdalena Pol-Szyszko, Beata Michalkiewicz

**Affiliations:** 1 Department of Catalytic and Sorbent Materials Engineering, Faculty of Chemical Technology and Engineering, West Pomeranian University of Technology in Szczecin, Piastów Ave. 42, 71-065 Szczecin, Poland; joanna.siemak@zut.edu.pl; 2 Faculty of Electrical Engineering, West Pomeranian University of Technology in Szczecin, 26 Kwietnia St. 10, 71-126 Szczecin, Poland; grzegorz.mikolajczak@zut.edu.pl; 3 Department of Plant Genetics, Breeding and Biotechnology, Faculty of Environmental Management and Agriculture, West Pomeranian University of Technology in Szczecin, 17 Słowackiego Str., 71-434 Szczecin, Poland; magdalena.pol-szyszko@zut.edu.pl

**Keywords:** sodium hydroxide, CO_2_ adsorption, avocado seed, activated carbons

## Abstract

The rise in atmospheric greenhouse gases like CO_2_ is a primary driver of global warming. Human actions are the primary factor behind the surge in CO_2_ levels, contributing to two-thirds of the greenhouse effect over the past decade. This study focuses on the chemical activation of avocado seeds with sodium hydroxide (NaOH). The influence of various preparation methods was studied under the same parameters: carbon precursor to NaOH mass ratio, carbonization temperature, and nitrogen flow. For two samples, preliminary thermal treatment was applied (500 °C). NaOH was used in the form of a saturated solution as well as dry NaOH. The same temperature of 850 °C of carbonization combined with chemical activation was applied for all samples. The applied modifications resulted in the following textural parameters: specific surface area from 696 to 1217 m^2^/g, total pore volume from 0.440 to 0.761 cm^3^/g, micropore volume from 0.159 to 0.418 cm^3^/g. The textural parameters were estimated based on nitrogen sorption at −196 °C. The XRD measurements and SEM pictures were also performed. CO_2_ adsorption was performed at temperatures of 0, 10, 20, and 30 °C and pressure up to 1 bar. In order to calculate the CO_2_ selectivity over N_2_ nitrogen adsorption at 20 °C was investigated. The highest CO_2_ adsorption (4.90 mmol/g) at 1 bar and 0 °C was achieved.

## 1. Introduction

The issue of global warming has been a worldwide priority for years, yet efforts to mitigate its effects remain insufficient. Recently, heightened attention to this issue has underscored that climate change, driven by greenhouse gas emissions, is raising alarm and making many people feel increasingly threatened [1,2]. Carbon dioxide is the most significant human-produced greenhouse gas, contributing to 77% of the anthropogenic greenhouse effect over the past decade, which translates to 26–30% of all CO_2_ emissions [3]. The primary source of human-related CO_2_ emissions is the burning of fossil fuels. The concentration of CO_2_ in flue gases varies depending on the type of fuel used; for example, coal results in 12–15 mol % CO_2_. In industries such as petroleum refining and other manufacturing processes, the CO_2_ levels in exhaust gases are process-dependent. For instance, oil refining emits 8–9 mol % CO_2_, cement production releases 14–33 mol % CO_2_, and iron and steel manufacturing results in 20–44 mol % CO_2_ [4].

There are multiple approaches to decreasing atmospheric CO_2_ emissions, such as the following: (a) improving the energy efficiency of fuels; (b) replacing fossil fuels with renewable energy sources like wind, solar, biomass, and geothermal power; (c) transitioning to technologies that use low-carbon energy sources such as natural gas; (d) reducing overall energy consumption; and (e) implementing pre-combustion, post-combustion, and oxy-combustion CO_2_ capture scenarios [5].

Additionally, more environmentally friendly and sustainable methods for CO_2_ capture have been explored, including the use of porous materials, especially carbon-based materials [6,7,8]. Carbon-based materials are renowned for their exceptional properties, such as adjustable and modifiable functionality, high surface area and total pore volume, especially micropore volume, broad temperature range for CO_2_ adsorption, rapid sorption kinetics, and excellent regeneration capabilities. In addition, these materials are cost-effective, industrially viable, and environmentally friendly [9,10]. They can be derived from a wide range of sources, and it is particularly worth highlighting that they can come from waste, especially from the food or agricultural industry [11,12].

Good CO_2_ sorbents prepared from a carbon source require chemical activation. KOH is mainly applied as the activation agent. Sometimes H_3_PO_4_, K_2_CO_3_, ZnCl_2_, and CaCl_2_ are also used to activate carbon production. NaOH is used very rarely in comparison to KOH. [11]. The reason is that usually activated carbons produced using NaOH showed much lower CO_2_ adsorption than activated carbons produced using KOH [13].

Nurfarhana et al. [14] used NaOH to treat natural rubber, converting it into activated carbon for CO_2_ adsorption. Natural rubber was carbonized at 400 °C for 2 h in a muffle furnace under a nitrogen flow. The pre-carbonized material was then mixed with solid NaOH at various ratios, ranging from 1:1 to 1:4 (natural rubber to NaOH). The activation process occurred in a tube furnace at 800 °C under a nitrogen atmosphere. Among all the samples, the highest specific surface area and total pore volume were achieved with a natural rubber to NaOH ratio of 1:4, reaching 1670 m^2^/g and 1.01 cm^3^/g, respectively. However, the CO_2_ adsorption for the mass ratio of 1:1 was relatively low, with a value of 0.69 mmol/g at 25 °C and 1 bar pressure. In contrast, the CO_2_ adsorption for the activated carbon obtained with a 1:4 ratio significantly increased to 2.98 mmol/g.

Dehkordi et al. [15] impregnated coal with NaOH solutions of varying concentrations (0.01–8 M) over different durations (4–7 h). Following impregnation, the coal samples were filtered and dried in an oven at 110 °C for 4–7 h. To examine the impact of washing on the activated carbon, some samples were immersed in deionized water. This study aimed to explore how different preparation parameters affect the surface properties and CO_2_ adsorption capacity of the activated carbon. The ideal conditions for good CO_2_ sorbent (51.4 mg/g) involved impregnation with a 1 M NaOH solution, followed by 6 h of mixing using a magnetic stirrer, without subsequent washing. The drying duration of the activated carbon had a minimal impact on its adsorption capacity.

Sodium hydroxide was used in the synthesis of sludge-activated carbon [16]. The carbon source was treated with a 5 wt % NaOH solution for 24 h. Following this, the reaction mixture was suction-filtered until the pH matched that of distilled water. Compared to the starting material, the specific surface area and pore volume of the sample treated with NaOH increased by 162% and 148%, respectively. The CO_2_ adsorption at a pressure of 4 MPa reached 65 cm^3^/g.

Solid monolithic graphene oxides (MGOs) were also used in the production of porous carbon using NaOH [17]. Graphene oxide (GO) was transformed into MGOs through a self-assembled reduction process at 90 °C, employing various weight ratios of oxalic acid (1:1, 1:0.500, and 1:0.250). Following this, the monoliths underwent carbonization at 600 °C and were chemically activated using different NaOH ratios (1:1, 1:2, and 1:3). 

Increasing the mass ratios of MGO to NaOH from 1:1 to 1:2 led to a surface area increase of approximately 2.6 times, ranging from 520.8 to 753.9 m^2^/g (the surface area of untreated MGO was 289.2 m^2^/g). As a result, this significantly boosted the CO_2_ capture capacity to 2.10 mmol/g at a temperature of 25 °C and pressure of 1 bar.

Table 1 compares the activated carbon prepared using NaOH and standard materials (graphene and silica gel), focusing on the preparation method, properties, and CO_2_ adsorption capacities, with published literature.

Nitrogen adsorption studies at −196 °C and carbon dioxide adsorption in the temperature range of 0–30 °C form the basis of our research. Therefore, we present the theoretical foundations about isotherms and hysteresis loops as outlined in the fundamental literature on this subject, recommended by IUPAC [21,22].

Physisorption isotherms can generally be categorized into six types [21]. Type I isotherms are characteristic of microporous solids and are concave to the p/p_0_ axis, approaching a limiting value as p/p_0_ approaches 1. Type II isotherms are typical of non-porous or macroporous adsorbents and are reversible. Type III isotherms are less common, convex to the p/p_0_ axis throughout, and occur when adsorbate–adsorbate interactions are significant. Type IV isotherms are associated with mesoporous materials and feature a hysteresis loop due to capillary condensation within the mesopores and a limiting uptake at high p/p_0_. The initial section of a Type IV isotherm is indicative of monolayer–multilayer adsorption. Type V isotherms resemble Type III but include a hysteresis loop, observed in certain porous adsorbents with weak adsorbent–adsorbate interactions. Finally, Type VI isotherms represent stepwise multilayer adsorption on a uniform non-porous surface, with the sharpness of the steps varying depending on the system and temperature.

In the 1985 IUPAC recommendations, physisorption isotherms were categorized into six types. However, in 2015, Type I was further subdivided into Types Ia and Ib. Type Ia is characterized by a very steep uptake at very low p/p_0_, whereas for Type Ib, the uptake at very low p/p_0_ is much milder. Type Ia isotherms are typical of microporous materials with predominantly narrow micropores (diameter smaller than 1 nm). In contrast, Type Ib isotherms are observed in materials with a broader pore size distribution, including wider micropores and possibly narrow mesopores (diameter smaller than 2.5 nm).

In the original 1985 IUPAC classification, four types of hysteresis loops were identified [21]. This classification was expanded to six types in 2015 [22]. The shape of hysteresis loops is closely linked to specific characteristics of the pore structure and the underlying adsorption mechanism.

In Type H1, the two branches are almost vertical and nearly parallel over a significant range of gas uptake. This loop is typically found in materials with a narrow range of uniform mesopores. Additionally, Type H1 hysteresis can appear in networks of ink-bottle pores where the neck size distribution is similar to the pore/cavity size distribution. Type H2(a) loops are characterized by a very steep desorption branch. This feature can be attributed to pore-blocking/percolation in a narrow range of pore necks or to cavitation-induced evaporation. Type H2(b) loops are also associated with pore blocking, but the neck size distribution is much broader. H2(a) loops are commonly found in silica gels, some porous glasses, and certain ordered mesoporous materials. The Type H3 loop resembles a Type II isotherm on the adsorption branch, and the lower limit of the desorption branch is usually at the cavitation-induced p/p_0_. This loop occurs in non-rigid aggregates of plate-like particles and in pore networks with macropores that are not completely filled with pore condensate. Type H4 loops have nearly horizontal and parallel branches over a wide range of p/p_0_. This loop is associated with the filling of micropores and is found in micro–mesoporous carbons. Type H5 loops are unusual and have a distinctive form associated with pore structures containing both open and partially blocked mesopores.

When using chemical activation, activated carbons are typically produced without temperature pretreatment. However, if temperature pretreatment is applied, authors do not compare these two methods. Similarly, the activating agent is commonly used in solution form, although the dry form is rarely used. No one has compared materials produced using the activating agent in both dry and dissolved forms until now.

The international market for avocado fruit and its derivates (primarily guacamole and avocado oil) has been growing rapidly in the last decade [23]. Only the avocado pulp is employed for commercial applications, while other fruit elements like the seed and peel have no practical use and are disposed of by landfilling. Avocado seeds, which represent up to 26 wt % of the fruit mass, are produced in large amounts in centralized avocado transformation plants. Nevertheless, the use of this carbon source for producing activated carbon has been described in only a few publications. Therefore, we decided to use avocado seeds for producing carbon materials for CO_2_ adsorption. Our team is the only one investigating the potential of such utilization of these activated carbons.

Our studies concerned five activated carbons obtained using the same temperature (850 °C) of activation combined with carbonization and an equal mass ratio of carbon source to activator (1:1). NaOH was utilized in two different forms: solid NaOH and its saturated solution. Activated carbons were produced with and without preliminary carbonization. We used the relatively uncommon activator NaOH. KOH is commonly used. The reason is that usually activated carbons produced using NaOH showed much lower CO_2_ adsorption than activated carbons produced using KOH. We demonstrate here that CO_2_ adsorption using NaOH can also be high if the appropriate method is applied. Avocado seeds were used as the carbon source. Avocado seeds as a carbon source have been described in only a few publications, and only our team has successfully produced activated carbons from avocado seeds for effective CO_2_ adsorption. The novelty of the work lies in comparing the obtained materials using dry NaOH and a saturated solution as the activating agent, as well as highlighting the significance of preliminary carbonization. We demonstrated the significant importance of both the form of NaOH and the use of preliminary carbonization. These factors influence both the properties of activated carbons and their CO_2_ adsorption capacity.

## 2. Materials and Methods

### 2.1. Materials and Reagents

We used the following chemicals for the synthesis of activated carbon from avocado seeds: NaOH (Chempur, Piekary Śląskie, Poland, pure p.a.) and 35–38% hydrochloric acid (Chempur, pure p.a.). The avocado seeds were dried at 60 °C for 24 h before undergoing pretreatment at 500 °C and chemical activation. After drying, the seeds were crushed and powdered using a Royal RCMZ-800 (Royal Catering expondo GmbH, Berlin, Germany) multi grinder. The powdered avocado seeds were then used for the production of activated carbon.

### 2.2. Activated Carbon Synthesis

**Figure 1:**
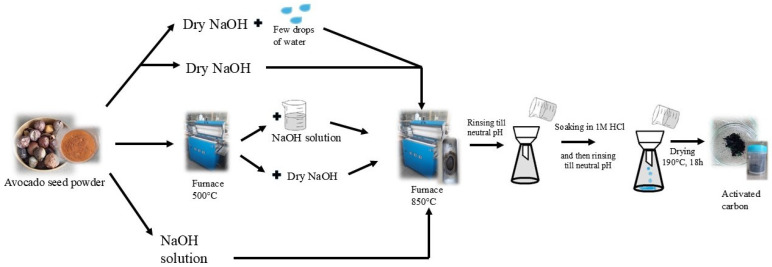
Activated carbon preparation from avocado seeds.

Five activated carbons were prepared from avocado seeds as described in Table 2 and shown in Figure 1.

In the case of the first two samples, an initial pretreatment of avocado seed was conducted at 500 °C. Subsequently, samples were mixed with a saturated solution of NaOH (C500_NaOH) or dry NaOH (C500_NaOHdry), followed by carbonization at a temperature of 850 °C for 1 h under nitrogen flow. They were then washed with 1 M HCl and rinsed with distilled water until reaching neutral pH. The two other samples were obtained as described above but without preliminary thermal treatment. The dried, powdered avocado seeds were mixed with a saturated solution of NaOH (C_NaOH) and dry NaOH (C_NaOHdry). The sample C_NaOHdry+H_2_O was obtained by mixing dried avocado seeds with dry NaOH and then adding some drops of water until the same pore volume of the starting materials was filled (incipient wetness method). In the incipient wetness method, only enough saturated NaOH solution is supplied to fill the pores of the starting materials. This minimizes the drying time of the sample, which reduces production costs. These costs are significantly lower than in the case of mixing the starting material with the saturated solution. In the latter method, the amount of saturated solution is such that the mass ratio of the carbon source to NaOH is 1. Here, we are dealing not only with the filling of the pores with the saturated solution.

### 2.3. Sample Characterization

The textural properties, including specific surface area (SSA), total pore volume (V_tot_), micropore volume (V_micro_), and pore size distribution in the range of 0.3 to 30 nm, were assessed using N_2_ sorption at −196 °C. These measurements were conducted using an automatic volumetric adsorption device, the ASAP Sorption Surface Area and Pore Size Analyzer (ASAP 2460, Micrometrics, Novcross, GA, USA). The specific surface area was determined using the Brunauer–Emmett–Teller (BET) theory. The BET equation is useful for determining the surface area of nonporous materials as well as those with macropores and mesopores (having sufficiently large pores) within the relative pressure range of p/p_0_ from 0.05 to 0.3. However, this method is not suitable for microporous adsorbents. Because the materials were mostly microporous, the method suggested by Rouquerol et al. [24] was applied. The procedure relies on two criteria: (a) the constant C must be positive, and (b) the use of the BET equation must be confined to the range where the term V(1-p/p_0_) consistently increases with p/p_0_. The relative range for surface area calculation was selected individually for each sample, also considering the value of the correlation coefficient, and ranged between 5 × 10^−5^ and 1 × 10^−2^ for microporous materials and ranged between 1 × 10^−2^ and 2 × 10^−1^ C_NaOHdry.

The total pore volumes were determined from the volume of nitrogen adsorbed at a relative pressure close to p/p_0_ ≈ 1. The pore size distribution and micropore volumes were estimated using the density functional theory (N_2_-DFT model for slit pores). Equilibrium adsorption isotherms of carbon dioxide at temperatures ranging from 0 to 30 °C and nitrogen at 20 °C were measured volumetrically using the ASAP Sorption Surface Area and Pore Size Analyzer (ASAP 2460, Micrometrics, Novcross, GA, USA), with pressures up to 1 bar. We select CO_2_ adsorption at a temperature range of 0–30 °C because this range is most popular in the investigations. We can easily compare our results with those of other researchers.

Phase analysis of the activated carbons was conducted using an X-ray diffractometer equipped with a Cu lamp by PANalytical Empyrean.

The average graphene sheet diameter (*La*) and average crystallite thickness (*L_c_*) were approximately calculated using the Scherrer equation [25]. Liu et al. [26] suggested K = 1.84 for the average graphene sheet diameter and K = 0.89 for the average crystallite thickness. The number of graphitic layers (*N*) was estimated using Equation (1):

(1) $$N=\frac{L_{c}}{d_{002}}$$

The structural morphology of the activated carbons was examined using ultra-high-resolution field–emission scanning electron microscopy (UHR FE-SEM, Hitachi SU8020 Hitachi Ltd., Tokyo, Japan). The powdered sample was mounted on the SEM stub using double-sided carbon tape. Images were captured with a 20 kV accelerating voltage using a triple detector system.

## 3. Results and Discussion

### 3.1. Materials Characterization

**Figure 2:**
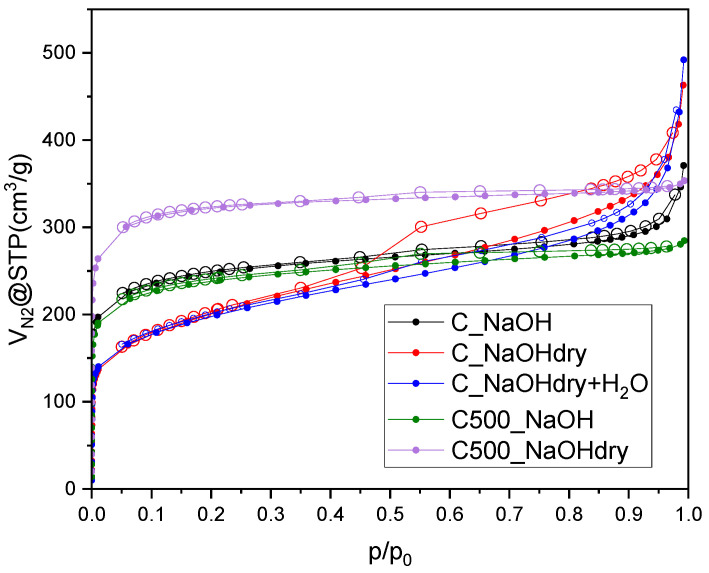
N_2_ sorption isotherms measured at −196 °C, adsorption brunch—filled symbols, desorption brunch—empty symbols.

The pore structure of activated carbons produced from avocado seeds was thoroughly investigated using N_2_ adsorption–desorption isotherms at −196 °C. As depicted in Figure 2, all adsorption isotherms exhibit a rapid N_2_ uptake at low pressure (relative pressure below 0.01), suggesting the presence of micropores in all the samples. The isotherms of samples pretreated at 500 °C and the sample prepared using NaOH solution show a sharp knee and a plateau after rapid uptake, indicating microporous materials. The isotherms of C500_NaOH and C500_NaOHdry correspond to a typical Type I isotherm according to the IUPAC classification [21], indicating that they are predominantly microporous. The very narrow hysteresis of H4 type is also present, indicating the presence of slit pores. For C_NaOH, we observed a mixed isotherm of Types I and II. This indicates the presence of micropores as well as macropores. For C_NaOH at higher relative pressure (>0.9), a significant increase in N_2_ adsorption is visible, which is evidence of the presence of macropores. The combined isotherms Types IV and II were identified for NaOHdry+H_2_O, C_NaOHdry. Type IV is characteristic of mesoporous materials. The very rapid uptake (Type II) at higher relative pressure (>0.9) indicates the presence of macropores. All the samples reveal hysteresis loops H4, which prove the presence of narrow slit-like pores. The hysteresis loop usually points to the presence of mesopores, but narrow hysteresis combined with a Type I isotherm indicates also microporosity.

**Figure 3:**
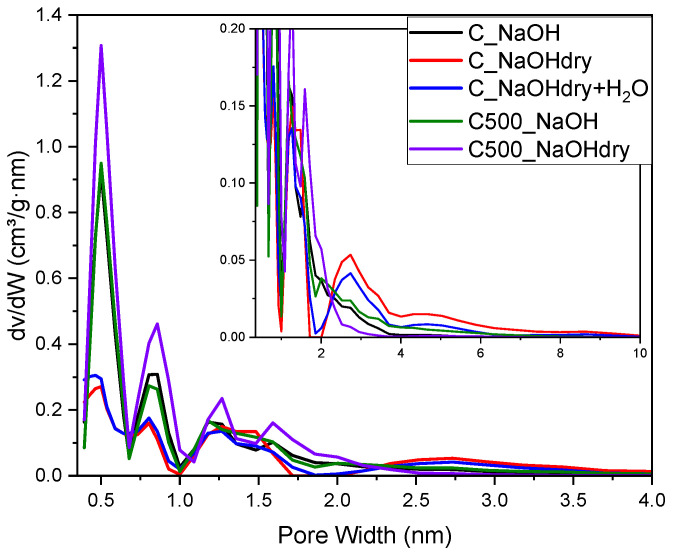
Pore size distribution of activated carbon.

The results of the pore size distribution calculations performed using the DFT method, presented in Figure 3, and the values of the textural parameters in Table 3 confirm the conclusions drawn from the shapes of the N_2_ adsorption isotherms. The pore size distribution curves (Figure 3) verify the microporous nature of C500_NaOHdry, C500_NaOH, and C_NaOH. The most microporosity resulted in significantly narrower and smaller ultramicropores (>0.7 nm). was observed for C500_NaOHdry. In contrast, C_NaOHdry and C_NaOHdry+H_2_O exhibited much wider and larger pores ranging from 2 to 4 nm.

Based on Table 3, it can be discerned that the obtained specific surface areas range from 696 to 1217 m^2^/g, total pore volume ranges from 0.440 to 0.761 cm^3^/g, micropore volume ranges from 0.159 to 0.418 cm^3^/g, and the ratio of the two mentioned parameters ranges from 22.08% to 76.42%. Despite using the same carbonization temperature and quantity of activating agent, the samples obtained differed according to the preparation methods used. The highest V_micro/tot_ values are observed in the carbons that underwent preliminary thermal treatment of the seeds: C500_NaOHdry (76.42%) and C500_NaOH (65.91%).

It has been found that using a saturated NaOH solution as an activator for dry avocado seeds is more advantageous for developing porosity compared to using dry NaOH. The material activated with a saturated NaOH solution possessed a high specific surface area and micropore volume. The use of dry NaOH and dry avocado seeds was decidedly unfavorable. The situation was reversed when the activation was preceded by an initial pretreatment at a temperature of 500 °C. Activation of the resulting char with a saturated NaOH solution allowed for the production of a material with porosity comparable to that of C_NaOH obtained without pretreatment. The textural parameters of C500_NaOH were even lower. On the other hand, the activation of char after the pretreatment with dry NaOH enabled the production of a highly porous material with high micropore volume and specific surface area.

Based on theoretical considerations, the possible reaction between NaOH, carbon, and byproducts can be proposed as follows:

4 NaOH + C → 4 Na + CO_2_ + 2 H_2_O
6 NaOH + 2 C → 2 Na + 3 H_2_ + Na_2_CO_3_
4 NaOH + 2 CO_2_ → 2 Na_2_CO_3_ + 2 H_2_O
Na_2_CO_3_ → Na_2_O + CO_2_
4 Na_2_O + C → 4 Na + CO_2_
4 NaOH + C → Na_2_CO_3_ + Na_2_O+ 2 H_2_
Na_2_CO_3_ +2 C→ 2 Na + 3 CO
C + CO_2_ ⇄ 2 CO

During the activation process, gases (H_2_, CO_2_, and CO), sodium, and sodium compounds (Na_2_O and Na_2_CO_3_). The reaction between the activating agent or byproducts and the carbon precursor leads to the breakdown of volatile organic compounds, resulting in the porous structure of the activated carbon samples. The gasification that occurs during activation with appropriate activating agents is crucial for the formation of pores. Thus, it can be argued that thermal treatment with an appropriate activating agent is essential for the formation of pores in activated carbon samples. During this process, the activating agent facilitates the development of a porous structure by promoting the removal of volatile substances and the creation of cavities. Without this step, the activated carbon would not exhibit the desired high surface area and porosity. Of course, both the choice of activating agent and the precise thermal conditions are crucial for producing high-quality activated carbon.

**Figure 4:**
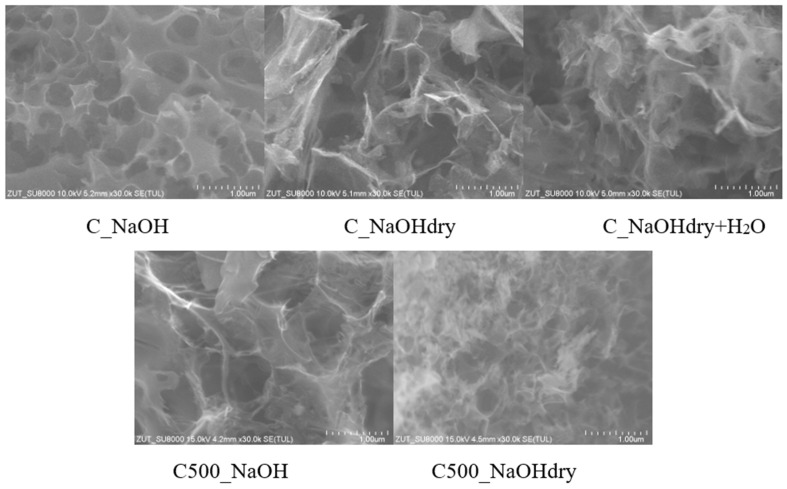
SEM images (30,000× magnification).

On the SEM images (Figure 4) of activated carbons with the highest specific surface area, such as C500_NaOHdry and C_NaOH, numerous macropores are observed, which, as commonly known, branch out into meso- and micropores within the material. For the material with the highest specific surface area (C500_NaOHdry), macropores have significantly smaller diameters on the surface than those in C_NaOH. In the case of other activated carbons with lower specific surface areas, the presence of thin, irregular planes creating occasional macropores with much larger sizes than in C_NaOH was observed. SEM images confirm the results obtained based on nitrogen adsorption measurements at a temperature of −196 °C.

**Figure 5:**
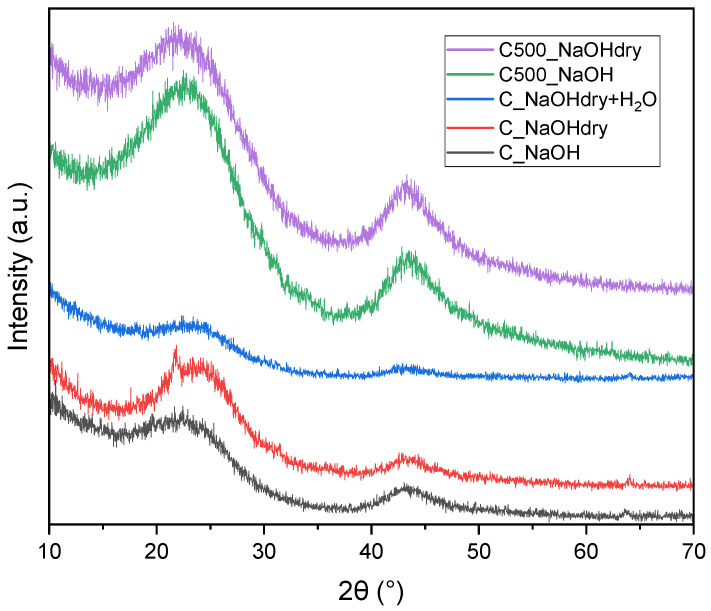
XRD pattern of activated carbons.

Figure 5 illustrates the XRD pattern of activated carbons activated by NaOH. Two broad peaks appearing around 22 and 44 degrees are observable. The shapes of these peaks suggest that the activated carbons derived from avocado seeds were predominantly amorphous.

The average crystallite thickness, number of graphitic layers, and average graphene sheet diameter calculated on the basis of XRD measurements are listed in Table 4. The average crystallite thickness of samples pretreated at 500 °C is the lowest, so the average number of layers in a stack is about 2. The average crystallite thickness of samples produced using dry NaOH without pretreatment is the highest. The average number of layers in a stack for these samples and for C_NaOH are about 3. The average graphene sheet diameters of samples produced using dry NaOH without pretreatment are the highest, while C_NaOH is the lowest. The results obtained from XRD studies are consistent with the porosity analysis. The greatest disorder (the smallest average graphene sheet diameter) was observed for materials with the highest specific surface area.

**Figure 6:**
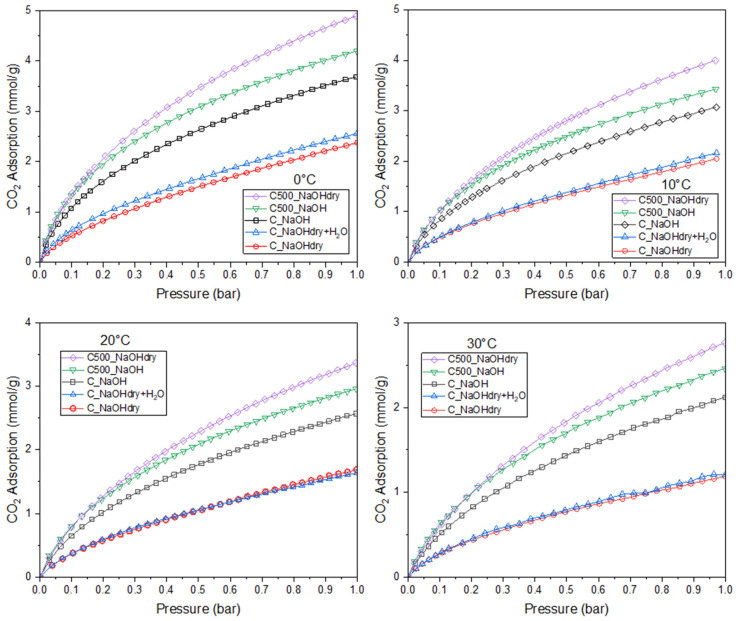
CO_2_ adsorption isotherms at different temperatures.

CO_2_ adsorption studies were conducted at four different temperatures: 0, 10, 20, and 30 °C and at a pressure of 1 bar. The results are presented in Table 5 and Figure 6.

Every sample exhibits a reduction in CO_2_ adsorption with rising temperature, which is attributed to physisorption.

**Figure 7:**
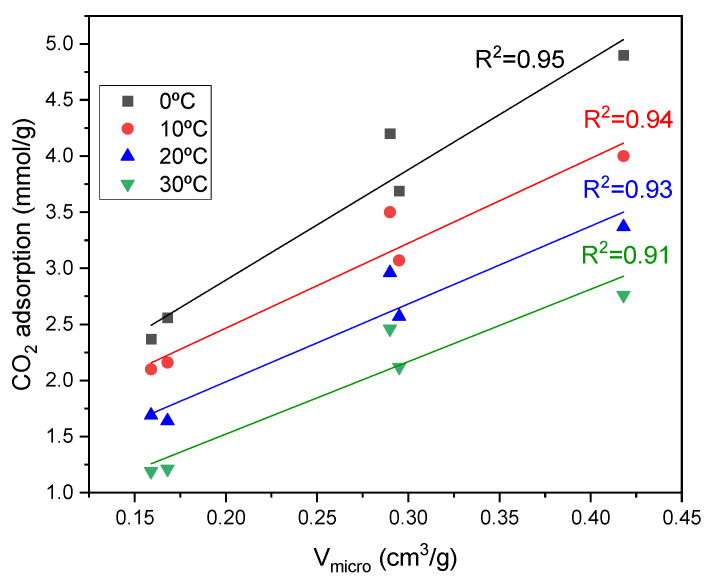
Correlation between CO_2_ adsorption and micropore volume.

The CO_2_ isotherms, measured up to 1 bar at temperatures of 0–30 °C, show that C500_NaOHdry performs best at pressure below 1 bar. These observations can be rationalized by noting that C500_NaOHdry has the largest volumes of micropores, which are effective in adsorbing CO_2_ at pressure below 1 bar. The lowest CO_2_ adsorption was observed in activated carbons with the smallest micropore volume (C_NaOHdry and C_NaOHdry+H_2_O). The relationship between CO_2_ adsorption and micropore volume for all temperatures is presented in Figure 7. The micropore volume exhibited a very close correlation with CO_2_ adsorption, having R^2^ values higher than 0.9. This outcome aligns with findings from recent research studies [27,28].

**Figure 8:**
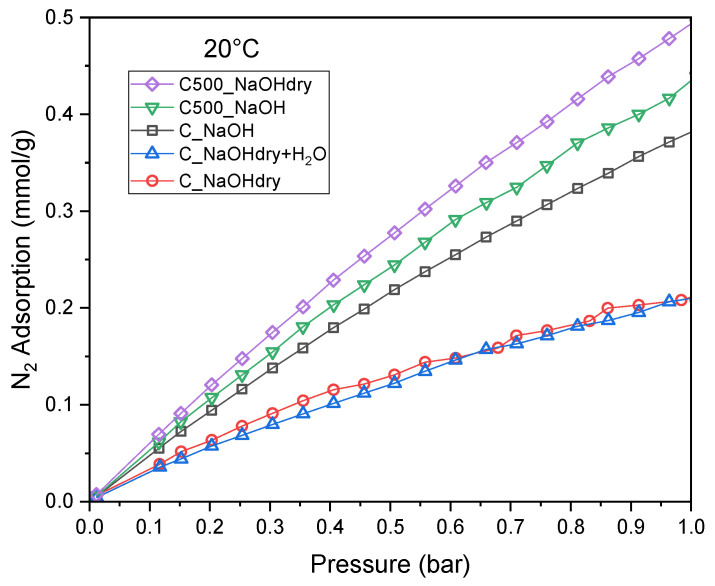
N_2_ adsorption isotherms.

The CO_2_ selectivity over N_2_ nitrogen adsorption at a temperature of 20 °C was calculated. The adsorption of nitrogen at a temperature of 20 °C was very low compared to the adsorption of CO_2_ at the same temperature (Figure 8).

Furthermore, the Ideal Adsorption Solution Theory (Equation (2)) was employed to calculate the theoretical selectivity of CO_2_ adsorption over N_2_ at a pressure of 1 bar and an equimolar composition. This approach is widely utilized to forecast the adsorption of individual components within gas mixtures based on data from single gas adsorption experiments.

(2) $$S_{IAST}=\frac{{q_{{CO}_{2}}@p}_{{CO}_{2}}/{q_{N_{2}}@p}_{N_{2}}}{p_{{CO}_{2}}/p_{N_{2}}}$$

where *q_j_* @ *p_i_* is the adsorption capacity of *i* at pressure *p_i_*.

For equimolar composition, Equation (2) simplifies to Equation (3).

(3) $$S_{{CO}_{2}/N_{2}}=\frac{q_{{CO}_{2}(p)}}{q_{N_{2}(p)}}$$

The results of selectivity of CO_2_ adsorption over N_2_ for equimolar composition (*S_eq_*) at 1 bar are presented in Table 5. The selectivity values are quite high and vary from 6.76 to 8.05.

Based on CO_2_ and N_2_ adsorption measurements, it is possible to determine the selectivity of CO_2_ adsorption over N_2_ using the IAST method across the entire pressure range. To achieve this, it is necessary to establish an adsorption model that best fits the experimental data. The adsorption model also allows for the calculation of the isosteric heat of CO_2_ adsorption, which is extremely important.

The two-parameter (Langmuir [29] and Freundlich [30]) and three-parameter (Sips [31], Toth [32], and Radke–Prausnitz [33]) models were employed to fit the experimental data. To evaluate the appropriateness of fitting isotherm models to the experimental data, the Hybrid Error Function (HYBRID) [34] was utilized. The sum of squared errors (SSE) is commonly employed to measure the disparity between observed data and the true mean. To enhance SSE at lower pressure values, each square of the error values was divided by the experimental CO_2_ adsorption value.

### 3.2. Studies on C500_NaOHdry as Potential CO_2_ Adsorbent

Among all the models, the Radke–Prausnitz model provided the highest accuracy in estimations for CO_2_ adsorption, with the Hybrid Error Function not higher than 0.001. For N_2_ adsorption, the best fit was ascertained for the Toth model with HYBRID equal to 0.00011. The constants of the Radke–Prausnitz and Toth models for the best adsorbent (C500_NaOHdry) are collected in Table 6.

The Radke–Prausnitz equation is formulated as follows:

(4) $$q=\frac{q_{mRP}\cdot b_{RP}\cdot p}{1+b_{RP}\cdot p^{n_{RP}}}[mmol/g]$$

*q_mRP_*—the maximum adsorption capacity [mmol/g];
*b_RP_*—the Radke–Prausnitz constant [bar^−1^];
*n_RP_*—Radke–Prausnitz model exponent.

Equation (5) outlines the Toth equation.

(5) $$q=\frac{q_{mT}b_{T}p}{(1+(b_{T}p{)}^{nT}{)}^{\frac{1}{nT}}}[mmol/g]$$

*q_mT_*—the maximum adsorption capacity [mmol/g];
*b_T_*—the Toth constant [bar^−1^];
*n_T_*—the heterogeneity factor.

**Figure 9:**
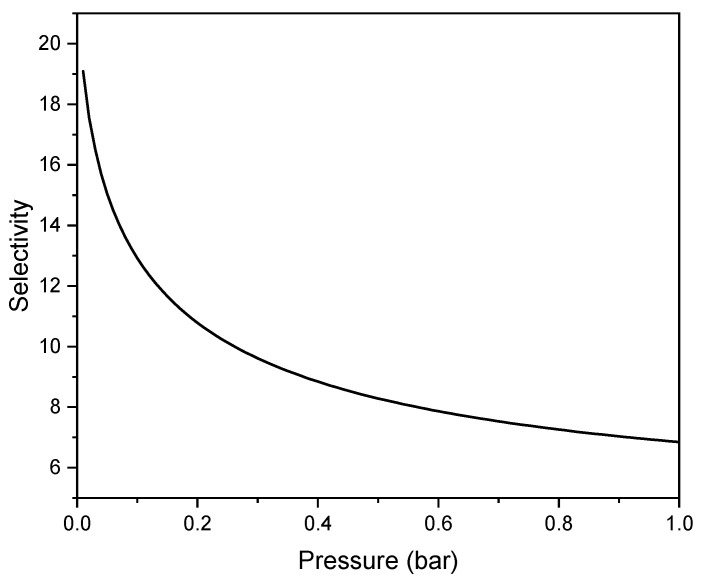
The CO_2_ selectivity over N_2_ for equimolar composition for the pressure up to 1 bar for C500_NaOHdry.

On the basis of the adsorption equilibrium models (2) and (3), the CO_2_ selectivity over N_2_ for equimolar composition at the pressure up to 1 bar for C500_NaOHdry was calculated and presented in Figure 9. The equimolar selectivity ranges from 19.17 to 6.84.

Post-combustion capture involves extracting CO_2_ from flue gas after combustion. Typically, the CO_2_ in flue gas is mixed with inert gases like nitrogen, argon, water vapor, and oxygen, resulting in a dilution of around 8–15% CO_2_. To determine if C500_NaOHdry is suitable as an adsorbent for CO_2_ removal from flue gas after combustion using the IAST method, the CO_2_ adsorption selectivity over N_2_ was calculated for CO_2_ contents of 8% and 15%. The selectivity for CO_2_ removal from flue gas is 16.65 and 13.48, respectively, which indicates that C500_NaOHdry is a potential sorbent for CO_2_ removal from flue gas.

The primary thermodynamic parameter of adsorption is the isosteric heat of adsorption. It can be determined from adsorption data at different temperatures by applying the Van’t Hoff or Clausius–Clapeyron Equation (6).

(6) $$Q_{iso}=-R{\left(\frac{\partial ln(p)}{\partial \left(\frac{1}{T}\right)}\right)}_{q}$$

**Figure 10:**
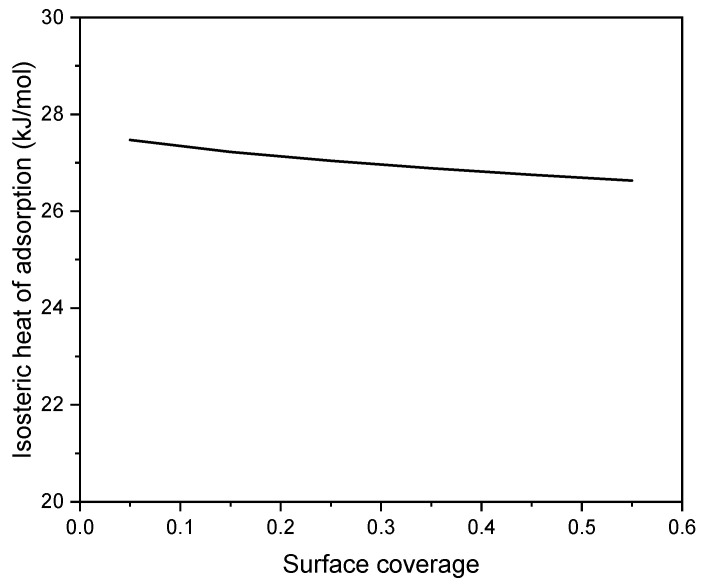
Isosteric heat of adsorption over C500_NaOHdry versus surface coverage.

In order to apply Equation (6), the Radke–Prausnitz Equation (4) along with the parameters listed in Table 6 were used to compute the pressure values for the five specified surface coverage levels. Since it is not possible to solve Equation (4) algebraically for pressure, numerical methods were utilized instead. By plotting ln(*p*) against the inverse of the absolute temperature (1/*T*) for each loading, straight lines were produced, each having a slope of −*Q_iso_*/*R*. This method enables the calculation of the isosteric heat of adsorption for the five specified surface coverage levels (Figure 10).

A high isosteric heat of adsorption generally indicates stronger interactions between the adsorbate and the adsorbent. The isosteric heat of adsorption values are below 50 kJ/mol, suggesting that CO_2_ adsorption over C500_NaOHdry samples is primarily of a physical nature. The isosteric heat of adsorption up to 0.5 surface coverage remains essentially constant, ranging from 27.4 to 26.6 kJ/mol. Such consistent values are highly favorable for CO_2_ sorbent. CO_2_ molecules are bound to the C500_NaOHdry sorbent with moderate strength, allowing for desorption without excessive energy input. Materials with a higher isosteric heat of adsorption than C500_NaOHdry will require more energy to desorb CO_2_, making the desorption process more costly. It is important to emphasize that the heat of adsorption on C500_NaOHdry is extremely close to the heat of liquefaction of CO_2_ (25 kJ/mol), which is the lower limit for the isosteric heat of adsorption. Therefore, this material ensures the lowest adsorption costs.

**Figure 11:**
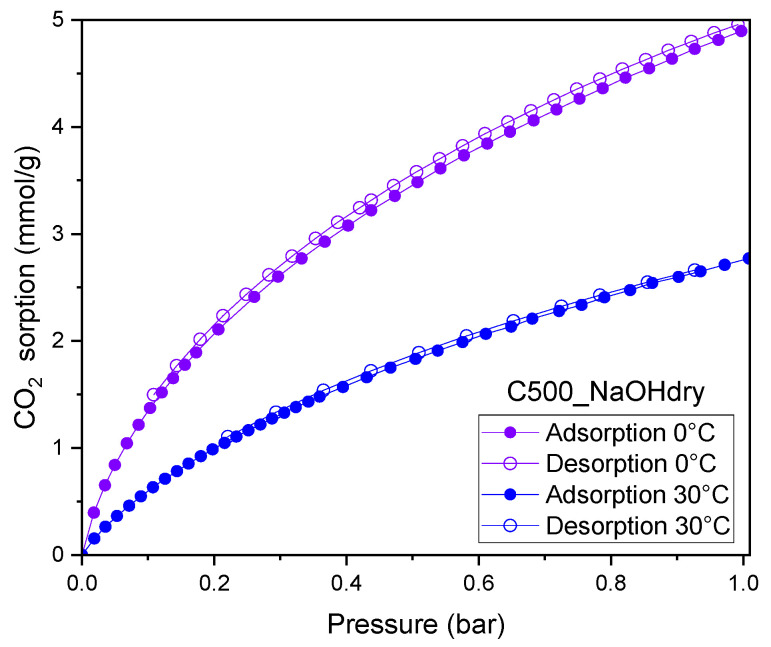
CO_2_ sorption isotherms measured at 10 and 30 °C for C500_NaOHdry.

Figure 11 shows adsorption–desorption isotherms at temperatures of 10 and 30 °C for C500_NaOHdry. The adsorption and desorption branches are nearly the same. The desorption isotherms lie slightly above the adsorption isotherms. Very narrow hysteresis is observed. The absorbed CO_2_ was completely released at low pressures, confirming the very weak interaction between CO_2_ and the sorbent [35].

## 4. Conclusions

This study focuses on the chemical activation of avocado seeds with NaOH. The influence of various preparation methods has been studied under the same parameters: carbon precursor to NaOH mass ratio, carbonization temperature, and nitrogen flow. We demonstrate here that CO_2_ adsorption using NaOH can be high if the appropriate method is applied.

The novelty of the work lies in comparing the obtained materials using dry NaOH and a saturated solution as the activating agent, as well as highlighting the significance of preliminary carbonization. We showed that preliminary carbonization at temperatures of 500 °C considerably improves CO_2_ uptake.

The significant importance of both the form of NaOH and the use of preliminary carbonization as parameters were demonstrated. The highest values of textural parameters and CO_2_ adsorption were achieved using thermal pretreatment and dry NaOH as the activating agent (C500_NaOHdry). Activation with NaOH enlarged the pores, resulting in an impressive specific surface area (1217 m^2^/g) and a significant micropore volume (0.547 cm^3^/g). This facilitated CO_2_ diffusion and adsorption (4.90 mmol CO_2_/g at 1 bar and 0 °C).

It was proven that the interaction between CO_2_ and C500_NaOHdry is very weak, and desorption is easy to achieve.

The equimolar CO_2_ selectivity over N_2_ was equal to 6.84, and for flue gas, it ranged from 16.65 to 13.48 depending on the CO_2_ content. The adsorbent exhibited quite low isosteric heat of adsorption, about 27 kJ/mol. All the values of selectivity and isosteric heat of adsorption indicate that C500_NaOHdry is a promising sorbent for CO_2_ capture from flue gas.

Of the various isotherm equations, the Radke–Prausnitz model proved effective in accurately predicting CO_2_ uptake isotherms.

In summary, the NaOH-activated carbon adsorbent exhibits potential for CO_2_ adsorption and separation applications, demonstrating impressive CO_2_ uptake and significant environmental benefits.

## Figures and Tables

**Table 1:** The activated carbons prepared using NaOH.

Sorbent	Method of Preparation	SSA (m^2^/g) V_tot_ (cm^3^/g)	q_CO_2___ (mmol/g)	t (°C) p (bar)	Ref.
AC from natural rubber	carbonized at 400 °C; mixed with solid NaOH at ratios ranging from 1:1 to 1:4 (natural rubber to NaOH); activation at 800 °C	1670 m^2^/g 1.01 cm^3^/g	2.98	25 1	[14]
AC from coal	impregnation with NaOH solutions concentrations (0.01–8 M), durations (4–7 h).	614 m^2^/g	1.68	25 2.12	[15]
AC from sludge	treating with a 5 wt % NaOH solution for 24 h; washing with water until the pH was neutral.	101 m^2^/g 0.123 cm^3^/g	2.90	30 40	[16]
AC from monolithic graphene oxides	mixed with NaOH at ratios ranging from 1 to 3; carbonized at 600 °C; washing with water until the pH was neutral.	754 m^2^/g 1.97 cm^3^/g	2.10	25 1	[17]
AC from waste tea	impregnation with solution of NaOH (mas ratio 1:1); carbonized at 600 °C; washing with water until the pH was neutral.	270 m^2^/g 0.106 cm^3^/g	0.96	25 1	[18]
Graphene-hydrazine	graphite oxide was treated by hydrazine hydrate at a temperature of 90 °C	409 m^2^/g 0.48 cm^3^/g	1.44	0 1	[19]
SBA-15 midified by BTESE	TEOS was replaced by 10% of 3-(triethoxysilyl) propylamine.	269 m^2^/g 0.36 cm^3^/g	1.21	30 1	[20]

**Table 2:** Procedures of sample preparation.

C500_NaOH	avocado seeds powder → furnace 500 °C → NaOH solution → furnace 850 °C
C500_NaOHdry	avocado seeds powder → furnace 500 °C → NaOH dry → furnace 850 °C
C_NaOH	avocado seeds powder +NaOH solution → furnace 850 °C
C_NaOHdry	avocado seeds powder +NaOH dry → furnace 850 °C
C_NaOHdry+H_2_O	avocado seeds powder +NaOH dry + few drops of H_2_O → furnace 850 °C

**Table 3:** Textural parameters of activated carbon.

AC	SSA	V_tot_	V_micro_	V_micro/tot_
	(m^2^/g)	(cm^3^/g)	(cm^3^/g)	(%)
C_NaOH	918	0.574	0.295	51.39
C_NaOHdry	700	0.716	0.159	22.21
C_NaOHdry+H_2_O	696	0.761	0.168	22.08
C500_NaOH	885	0.440	0.290	65.91
C500_NaOHdry	1217	0.547	0.418	76.42

**Table 4:** The average crystallite thickness (*L_c_*), number of graphitic layers (*N*), and average graphene sheet diameter (*L_a_*).

AC	*L_c_*	*N*	*L_a_*
	(nm)		(nm)
C_NaOH	1.04	2.71	2.39
C_NaOHdry	1.23	3.27	4.10
C_NaOHdry+H_2_O	1.23	3.30	3.69
C500_NaOH	0.88	2.29	3.45
C500_NaOHdry	1.04	2.71	2.39

**Table 5:** CO_2_ and N_2_ adsorption values over activated carbon.

AC	*q*_CO_2__0 °C_	*q*_CO_2__10 °C_	*q*_CO_2__20 °C_	*q*_CO_2__30 °C_	*q*_N_2__20 °C_	*S_eq_*
	(mmol/g)	(mmol/g)	(mmol/g)	(mmol/g)	(mmol/g)	
C_NaOH	3.69	3.07	2.57	2.12	0.38	6.76
C_NaOHdry	2.37	2.10	1.69	1.19	0.21	8.05
C_NaOHdry+H_2_O	2.56	2.16	1.64	1.21	0.21	7.81
C500_NaOH	4.20	3.50	2.96	2.46	0.37	8.00
C500_NaOHdry	4.90	4.00	3.37	2.76	0.50	6.84

**Table 6:** The constants of Radke–Prausnitz and Toth models for C500_NaOHdry.

Radke–Prausnitz Model for CO_2_					Toth Model for N_2_	
	Temperature [°C]					
	0	10	20	30		20
*q_mRP_*	5.76	5.01	4.39	3.83	*q_mT_*	3.01
*b_RP_*	5.81	4.39	3.33	2.61	*b_T_*	0.21
*n_RP_*	0.59	0.59	0.59	0.6	*n_T_*	0.90
HYBRID	0.00103	0.00078	0.00028	0.00081	HYBRID	0.00011

## Data Availability

The raw data supporting the conclusions of this article will be made available by the authors upon request.
